# Overexpression of GUCY1A2 Correlates With Poor Prognosis in Gastric Cancer Patients

**DOI:** 10.3389/fonc.2021.632172

**Published:** 2021-05-25

**Authors:** Xin Li, Xiaowei Chen, Xueju Hu, Yan Shen, Rui Xu, Leilei Wu, Xiaobing Shen

**Affiliations:** ^1^ Key Laboratory of Environmental Medicine Engineering, Ministry of Education, School of Public Health, Southeast University, Nanjing, China; ^2^ Department of Epidemiology and Health Statistics, School of Public Health, Southeast University, Nanjing, China

**Keywords:** gastric cancer, GUCY1A2, TCGA, GSEA, prognosis

## Abstract

**Background:**

Nitric oxide (NO) and cyclic guanosine phosphate (cGMP) play important roles in blood pressure regulation, neurotransmitter delivery, renal function, and tumorigenesis and development. The intermediate link of this signaling pathway, soluble guanylyl cyclase (sGC), is particularly important. However, the role of the GUCY1A2 gene encoding the sGC α2 subunit is unknown.

**Methods:**

Gene expression and clinical data were obtained from The Cancer Genome Atlas (TCGA) database. After screening for GUCY1A2 expression, the expression differences between gastric cancer (GC) tissues and adjacent noncancerous tissues were determined using R software. Quantitative real-time polymerase chain reaction (qRT-PCR) and meta-analysis were used to verify the result. The correlation between the expression of GUCY1A2 and clinicopathological parameters was explored by logistic regression. Then, Kaplan-Meier survival analysis and the Cox proportional hazards regression were used to evaluate the relationship between the expression of GUCY1A2 and the survival of GC patients. Finally, gene set enrichment analysis (GSEA) was used to explore and analyze the GC-related signaling pathways affected by high GUCY1A2 expression.

**Results:**

We found that GUCY1A2 was highly expressed in GC tissues compared to adjacent noncancerous tissues (*P* < 0.001). qRT-PCR (*P* < 0.001) and meta-analysis (SMD = 0.65, 95% CI: 0.20-1.10) confirmed the difference in GUCY1A2 expression. Logistic regression analysis showed that high expression of GUCY1A2 was associated with histological grade (OR=1.858 for poor vs. well or moderate, *P* = 0.004) and T stage (OR = 3.389 for T3 vs. T1, *P* = 0.025; OR = 3.422 for T4 vs. T1, *P* = 0.028). Kaplan-Meier curves indicated that GC patients with high expression of GUCY1A2 had a poor prognosis than that of patients with low expression. Univariate analysis indicated that GUCY1A2 and some clinicopathological parameters, such as age, pathological stage, and TNM stage, may predict poor prognosis. Multivariate analysis further confirmed that GUCY1A2 was an independent prognostic marker (HR = 1.699; 95%CI, 1.175-2.456; *P* = 0.005). GSEA showed that the high GUCY1A2 phenotype is significantly enriched for tumor-associated signaling pathways.

**Conclusions:**

GUCY1A2 is highly expressed in GC and may be used as a potential prognostic marker.

## Introduction

Gastric cancer (GC) is a malignant tumor with high morbidity and mortality rates worldwide. Its incidence rate and fatality rate are fifth and third, respectively. This disease has become a major public health problem that seriously threatens human health. In 2018, there were approximately 1, 000, 000 new cases and 783, 000 deaths (1). Moreover, the incidence rate of GC in Eastern Asia has increased notably. Although the treatment of GC has shown major progress, treatment strategies for this disease are still limited. In particular, patients with advanced GC can only undergo palliative tumor reduction surgery or other conservative treatments. Therefore, it is important to identify prognostic biomarkers of GC.

Guanylate cyclase is an enzyme that catalyzes the conversion of guanosine triphosphate (GTP) to cyclic guanosine monophosphate (cGMP). The guanylate cyclase C (GC-C) receptor is present in intestinal epithelial cells, can increase cGMP levels by binding with enterotoxin and participates in many important physiological processes of cells (2). Because of its important physiological functions, GC-C has become a therapeutic target for gastrointestinal disorders and colorectal cancer (3, 4). Two other guanylate cyclase isoforms, GC1 and GC2, encoded by GUCY2D and GUCY2F, are related to visual function (5). The soluble guanylate cyclase (sGC) is a heterodimeric enzyme composed of α (α1, α2) and β (β1, β2) subunits (6, 7). The α and β subunits of sGC are encoded by different genes and can be regulated independently in most human tissues (8). sGC, as a major receptor for nitric oxide (NO), generates cyclic guanosine monophosphate (cGMP), which is involved in various physiological activities (9, 10), such as platelet aggregation (10), smooth muscle relaxation (11, 12) and neurotransmitter delivery. In cancer, the NO/sGC/cGMP signaling pathway plays a dual role. On the one hand, it increases the frequency of mutations in the tumor suppressor gene P53 thereby promoting tumor development, and on the other hand it may mediate the apoptotic effects of cancer cells affecting the occurrence and development of tumor (13–15). The α1β1 isoform is the most active and studied type in sGC (16). Some studies have shown that sGCα1 expression is upregulated in breast and prostate cancers (17, 18). However, the expression of sGCα1 decreased significantly in astrocytoma, oligodendrocytoma, and glioblastoma multiforme (19). The sGCβ1 subunit may affect cancer progression by regulating gene expression and chromatin remodeling (20). The above research indicates that the subunits of sGC play an important role in tumorigenesis and development, and this study focused on the GUCY1A2 gene, which encodes the α2 subunit of sGC. One study reported sequencing analysis of a pediatric lung adenocarcinoma presenting with brain metastasis revealed a mutation in GUCY1A2 (21). This suggests that the GUCY1A2 gene may be involved in the process of tumor development, but few related studies have been reported. Whether the GUCY1A2 gene can be used as a prognostic marker or therapeutic target for tumors remains to be further explored.

Using the Cancer Genome Atlas (TCGA) database, we downloaded and analyzed gene and clinical data, followed by Gene Expression Omnibus (GEO) dataset analysis and quantitative real-time polymerase chain reaction (qRT-PCR) to validate the analysis results preliminarily. The prognostic value of GUCY1A2 in GC was evaluated by Kaplan-Meier survival curve and Cox regression analysis. Through the above methods we explored the expression and prognostic significance of the GUCY1A2 gene encoding the α2 subunit in GC. Our results provide certain basis for GUCY1A2 as a promising prognostic marker for GC.

## Materials and Methods

### Datasets and Clinical Specimens

The gene expression data (407 cases, workflow type: RNASeq-FPKM) and clinical information (443 cases) for this study were obtained from the TCGA dataset (https://portal.gdc.cancer.gov/). As of March 2020, we included gene expression data on GUCY1A2 from 375 GC tissue samples and 32 adjacent noncancerous tissue samples, as well as the clinical data of patients such as age, gender, pathological stage, histological grade, TNM stage, survival time and survival outcome. The detailed clinicopathological parameters are shown in **Table S1**. In addition, we collected 51 pairs of GC tissues and adjacent noncancerous tissues from Zhongda Hospital of Southeast University and approved by the Ethics Committee of Zhongda Hospital, Southeast University. These samples were obtained from patients who had never received preoperative radiotherapy or chemotherapy before surgical resection. The samples were collected and stored in RNA later (Austin, Texas, USA) at -80°C until utilized.

### RNA Extraction and Quantitative Real-time Polymerase Chain Reaction (qRT-PCR) Analysis

Total RNA from GC tissues and adjacent noncancerous tissues was extracted using TRIzol reagent (Invitrogen, Carlsbad, USA). Then, the concentration and purity of total RNA were measured with NanoDrop 2000 spectrophotometer (Thermo Fisher Scientific, Waltham, USA). Reverse transcription was performed using PrimeScript™ RT kit (Takara, Tokyo, Japan). The reaction conditions of the PCR system according to 2x RealStar SYBR Mixture kit (with ROX) instruction on a StepOnePlus PCR system (Applied Biosystems, Waltham, USA) were as follows: predenaturation at 95°C for 2 min and then 95°C for 15 seconds, 60°C for 30 seconds, and 72°C for 30 seconds, for a total of 40 cycles. The forward primer of GUCY1A2 is TTGGATGAACTCATGGGCCG, and the reverse primer is TCAACCCATCTTGGGCCTTT. The primer sequence of β-actin used for qPCR was as follows: forward: TCCATCATGAAGTGTGACGT, reverse: GAGCAATGATCTTGATCTTCAT. We used β-actin as an internal control and compared the mRNA expression levels by the 2^-ΔΔCt^ method.

### Verification of GUCY1A2 by the GEO Datasets

By using “cancer”, “tumor”, “carcinoma” or “neoplasm” and “gastric” or “stomach” as search terms and “*Homo sapiens*” as qualifier, we searched and screened microarray and RNA sequencing data from the GEO database. We downloaded a total of 11 eligible datasets (GSE13195, GSE13911, GSE26899, GSE27342, GSE29272, GSE33335, GSE37023, GSE54129, GSE63089, GSE64591 and GSE65801) (**Table S2**). A comprehensive meta-analysis was conducted to verify the differences in GUCY1A2 expression by Review Manager 5.3. The standard mean deviation (SMD) and 95% confidence interval (CI) were used to calculate the combined value. The *χ^2^* and *I^2^* statistical test were used to evaluate the heterogeneity between the included data sets. When *P* > 0.05 or *I^2^* < 50%, the combined effect was calculated by the fixed effects model; otherwise, the random effects model (*P* < 0.05 or *I^2^* > 50%) was used.

### Gene Set Enrichment Analysis (GSEA)

According to the median expression of GUCY1A2, GC patients were divided into two groups (high expression group and low expression group). GSEA was used to investigate the potential mechanism of the expression of GUCY1A2 as a prognostic factor for GC. The annotated gene set was selected (c2.cp.kegg.v6.2.symbols.gmt) as the reference gene set. 1,000 gene sets were arranged in each analysis, and gene set permutations were performed 1,000 times for each analysis. The normalized enrichment score (NES), nominal *P*-value, and false discovery rate (FDR) Q-value were used to estimate the significantly enriched gene sets.

### Statistical Analysis

All statistical analyses were performed with R 3.6.3 software, and *P* < 0.05 was considered statistically significant. First, we compared the expression of GUCY1A2 in GC tissues and adjacent noncancerous tissues *via* the Wilcoxon rank-sum test. Second, the Kaplan-Meier method was used to analyze the correlation between the expression level of GUCY1A2 and the overall survival (OS) of patients. The relationship between the expression of GUCY1A2 and clinicopathological parameters was analyzed by logistic regression. Then, the correlation of clinicopathological parameters and GUCY1A2 expression with OS was analyzed using univariate Cox regression analysis, and multivariate Cox regression analysis was performed to further verify whether the above possible prognostic factors were independent.

## Results

### GUCY1A2 Was Highly Expressed in GC Tissues

**Figure 1:**
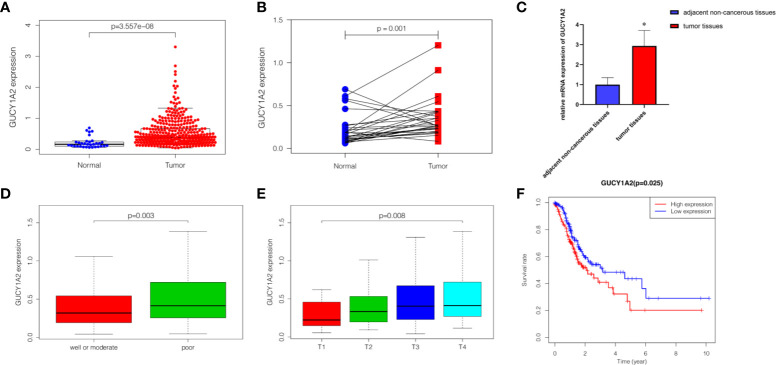
The expression of GUCY1A2 and its association with clinicopathological parameters and OS based on TCGA database. **(A)** GUCY1A2 expression was higher in GC tissues than in adjacent noncancerous tissues (*P* < 0.001); **(B)** GUCY1A2 was expressed at higher levels in GC tissues compared to 27 paired adjacent noncancerous tissues (*P* = 0. 001); **(C)** qRT-PCR analysis of GUCY1A2 expression in 51 pairs of GC and adjacent noncancerous tissue samples (*P* < 0.001); **(D)** Correlation between GUCY1A2 expression and histological grade (*P* = 0.003); **(E)** Correlation between GUCY1A2 expression and T stage (*P* = 0.008); **(F)** Kaplan-Meier curve of the relationship between GUCY1A2 expression and OS of GC patients (*P* = 0.025). OS, overall survival; TCGA, The Cancer Genome Atlas; GC, gastric cancer; qRT-PCR, quantitative real-time polymerase chain reaction. *P < 0.05.

The Wilcoxon rank-sum test was used to compare the expression of GUCY1A2 in 375 GC tissues and 32 adjacent noncancerous tissues. We discovered that the expression of GUCY1A2 in GC tissues was significantly higher than that in adjacent noncancerous tissues (*P* < 0.001) (**Figure 1A**). In addition, in 27 pairs of GC and adjacent noncancerous tissues, GUCY1A2 was also overexpressed in GC tissues compared with adjacent noncancerous tissues (*P* = 0.001) (**Figure 1B**). In summary, GUCY1A2 was highly expressed in GC tissues.

### Verification of GUCY1A2 Upregulation by qRT-PCR and Meta-analysis

**Figure 2:**
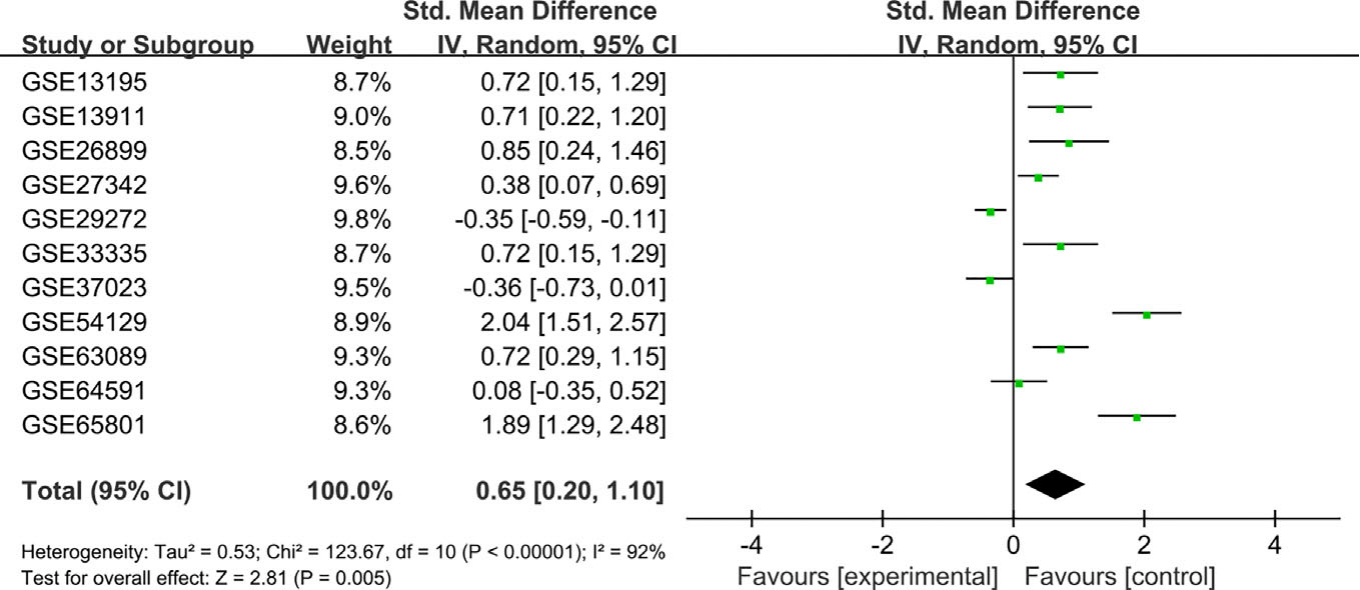
Meta-analysis of GUCY1A2 expression data from GEO microarrays. The pooled SMD of GUCY1A2 was 0.65 (95%CI: 0.20-1.10) by the random effects model. GEO, Gene Expression Omnibus; SMD, standard mean difference; CI, confidence interval.

To verify the difference in GUCY1A2 expression in TCGA database, we used qRT-PCR to evaluate the expression of GUCY1A2 at the transcriptional level. We found that the GUCY1A2 mRNA level in GC tissues was significantly higher than that in adjacent noncancerous tissues (*P* < 0.001, **Figure 1C**). In addition, a comprehensive meta-analysis of GUCY1A2 expression data for patients with GC in GEO database was conducted (**Table S2**). The results further confirmed the differential expression of GUCY1A2 in GC tissues and adjacent noncancerous tissues (SMD=0.65, 95%CI: 0.20-1.10, **Figure 2**).

### Correlations Between GUCY1A2 Expression and Clinicopathological Parameters of GC Patients

To probe the relationship between the expression of GUCY1A2 and the clinicopathological parameters of the GC patients, we used R software to further analyze the expression level of GUCY1A2 in GC patients with different clinicopathological parameters. **Figure 1D** showed that the expression of GUCY1A2 in the poor group (G3) was higher than that in the well or moderate group (G1/2) for histological grade (*P* = 0.003). In addition, as the T stage increased, the expression of GUCY1A2 was also elevated (**Figure 1E**, *P* = 0.008). These results indicated that GUCY1A2 may function as an oncogene. Logistic regression analysis with GUCY1A2 expression as a categorical dependent variable showed that increased GUCY1A2 expression was significantly associated with histological grade (OR=1.858 for poor vs. well or moderate, *P* = 0.004) and T stage (OR = 3.389 for T3 vs. T1, *P* = 0.025; OR = 3.422 for T4 vs. T1, *P* = 0.028) (**Table 1**).

**Table 1:** Relationships between GUCY1A2 expression and clinicopathological parameters of GC patients.

Clinicopathological parameters	Total (N)	Odds ratio in GUCY1A2 expression	*p*-Value
Age			
<60 *vs*. ≥60	371	0.729 (0.466-1.137)	0.165
Gender			
Male *vs*. Female	371	0.903 (0.592-1.379)	0.639
Tumor differentiation			
Poor *vs*. Well or moderate	366	1.858 (1.219-2.846)	**0.004**
Pathological stage			
Stage II *vs*. Stage I	164	1.198 (0.620 -2.339)	0.593
Stage III *vs*. Stage I	203	1.699 (0.905 -3.232)	0.101
Stage IV *vs*. Stage I	91	1.741 (0.754 - 4.081)	0.196
T stage			
T2 *vs*. T1	99	1.771 (0.610-5.922)	0.315
T3 *vs*. T1	187	3.389 (1.235-10.875)	**0.025**
T4 *vs*. T1	119	3.422 (1.208-11.251)	**0.028**
Lymph node metastasis			
Positive *vs*. Negative	357	1.073 (0.685-1.682)	0.758
Distant metastasis			
M1 *vs*. M0	355	1.304 (0.577-3.020)	0.525

Bold values indicate P<0.05.

### Kaplan-Meier Estimate of Survival in GUCY1A2-High and GUCY1A2-Low Patients

**Figure 3:**
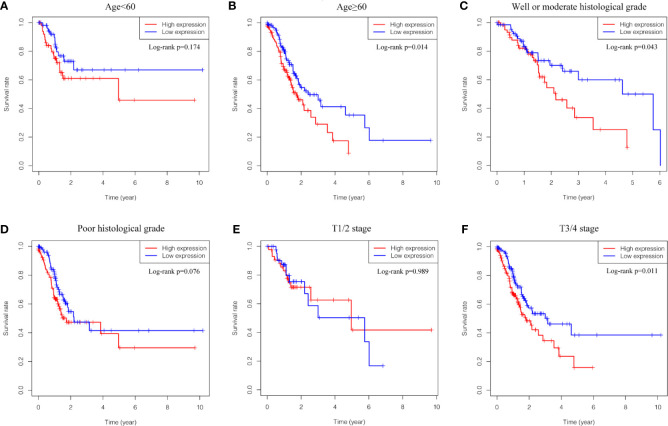
Relationship between GUCY1A2 expression and OS in different subgroups of clinicopathological parameters. **(A)** OS curve of GC patients with age<60 (*P* = 0.174); **(B)** OS curve of GC patients with age≥60 (*P* = 0.014); **(C)** OS curve of GC patients with well or moderate histological grade (*P* = 0.043); **(D)** OS curve of GC patients with poor histological grade (*P* = 0.076); **(E)** OS curve of GC patients with T1/2 stage (*P* = 0.989); **(F)** OS of GC patients with T3/4 stage (*P* = 0.011). OS, overall survival; GC, gastric cancer.

**Figure 4:**
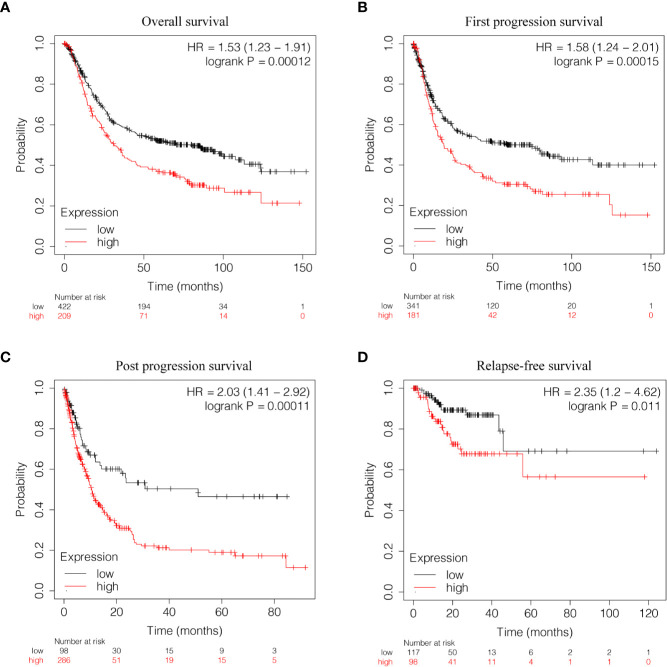
Relationship between GUCY1A2 expression and survival of GC patients based on Kaplan-Meier Plotter database. **(A)** OS curve of GC patients (*P* < 0.001); **(B)** FP curve of GC patients (*P* < 0.001); **(C)** PPS curve of GC patients (*P* < 0.001); **(D)** RFS curve of GC patients (*P* = 0.011). OS, overall survival; FP, first progression survival; PPS, post progression survival; RFS, relapse-free survival.

Kaplan-Meier survival analysis was utilized to evaluate the prognosis of GC patients with different levels of GUCY1A2 from TCGA database. The results indicated that the high GUCY1A2 expression group had a poor prognosis than the low GUCY1A2 expression group (*P* = 0.025) (**Figure 1F**). We further analyzed the correlation between GUCY1A2 expression and OS in GC patients with different clinicopathological parameters to investigate the prognostic value of GUCY1A2. The subgroup analysis showed that the OS was significantly different when grouped by GUCY1A2 for age≥60 years (*P =* 0.014), well or moderate status (*P =* 0.043) and T3/4 stage (*P =* 0.011). In other words, the OS was poor in patients with high GUCY1A2 expression and an age ≥60 years, well or moderate status and T3/4 stage (**Figures 3A–F**). The relationship between GUCY1A2 expression level and survival rate of GC patients was analyzed using the Kaplan-Meier Plotter database. The results showed that GUCY1A2 expression in GC patients was associated with OS (HR = 1.53; 95%CI, 1.23-1.91, *P* < 0.001), first progression survival (FP) (HR = 1.58; 95%CI, 1.24-2.01, *P* < 0.001), post progression survival (PPS) (HR = 2.03; 95%CI, 1.41-2.92, *P* < 0.001) and relapse-free survival (HR = 2.35; 95%CI, 1.20-4.62, *P* = 0.011) (**Figures 4A–D**). These results suggest that high expression of GUCY1A2 is associated with poor prognosis in GC patients.

### Prognostic Significance of GUCY1A2 Expression in GC patients

**Figure 5:**
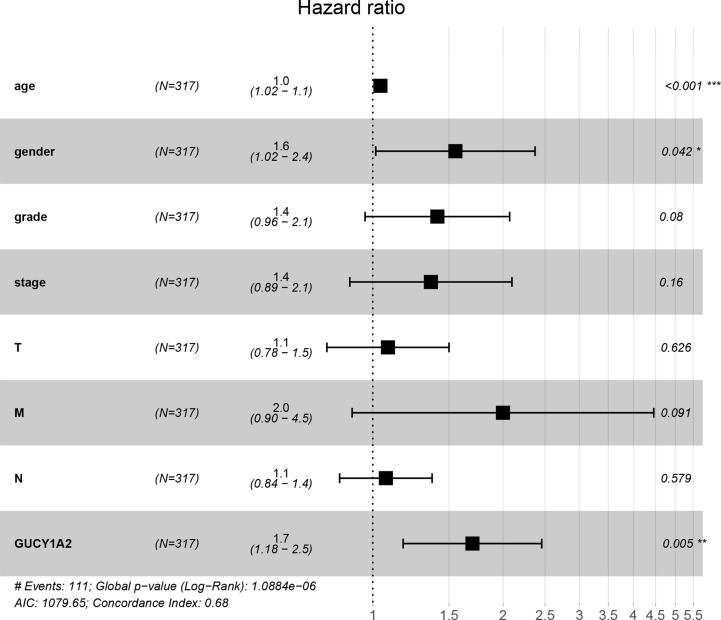
Forest plot of the multivariate Cox regression model. GUCY1A2 was an independent predictor of poor survival rate (HR = 1.699; 95%CI, 1.175-2.456; *P* = 0.005). HR, hazard ratio; CI, confidence interval. *P < 0.05, **P < 0.01, ***P < 0.001.

To further explore the GUCY1A2 expression related to the prognosis of GC, we conducted univariate analysis. The results demonstrated that high GUCY1A2 expression (HR = 1.433; 95%CI, 1.030-1.992; *P* = 0.032) and other clinicopathological parameters, such as age (HR = 1.027; 95%CI, 1.008-1.046; *P* = 0.006), pathological stage (HR= 1.535; 95%CI, 1.221-1.931; *P* < 0.001), T stage (HR = 1.298; 95%CI, 1.023-1.645; *P* = 0.032), N stage (HR = 1.267; 95%CI, 1.069-1.502; *P* = 0.006), and M stage (HR = 2.048; 95%CI, 1.096-3.827; *P* = 0.025), were associated with poor OS (**Table 2**). Multivariate analysis was performed to confirm the prognostic value of GUCY1A2 expression. The results showed that age (HR = 1.042; 95%CI, 1.021-1.063; *P* < 0.001), gender (HR = 1.552; 95%CI, 1.016-2.370; *P* = 0.042) and GUCY1A2 expression (HR = 1.699; 95%CI, 1.175-2.456; *P* = 0.005) were independently associated with OS (**Table 2**) (**Figure 5**). In summary, the expression of GUCY1A2 is an independent prognostic factor, and increased GUCY1A2 levels are associated with poor OS.

**Table 2:** Univariate and multivariate analysis of prognostic factors in GC patients.

Parameter	Univariate analysis			Multivariate analysis		
	HR	95%CI	*P*	HR	95%CI	*P*
Age	1.027	1.008-1.046	**0.006**	1.042	1.021-1.063	**0.000**
Gender	1.484	0.980-2.247	0.062	1.552	1.016-2.370	**0.042**
Grade	1.368	0.947-1.977	0.095	1.410	0.960-2.071	0.080
Pathological stage	1.535	1.221-1.931	**0.000**	1.362	0.885-2.097	0.160
T	1.298	1.023-1.645	**0.032**	1.084	0.784-1.500	0.626
N	1.267	1.069-1.502	**0.006**	1.072	0.838-1.371	0.579
M	2.048	1.096-3.827	**0.025**	1.998	0.896-4.457	0.091
GUCY1A2	1.433	1.030-1.992	**0.032**	1.699	1.175-2.456	**0.005**

Bold values indicate P<0.05. HR, hazard ratio; CI, confidence interval.

### Identification of GUCY1A2-Related Signaling Pathways

**Figure 6:**
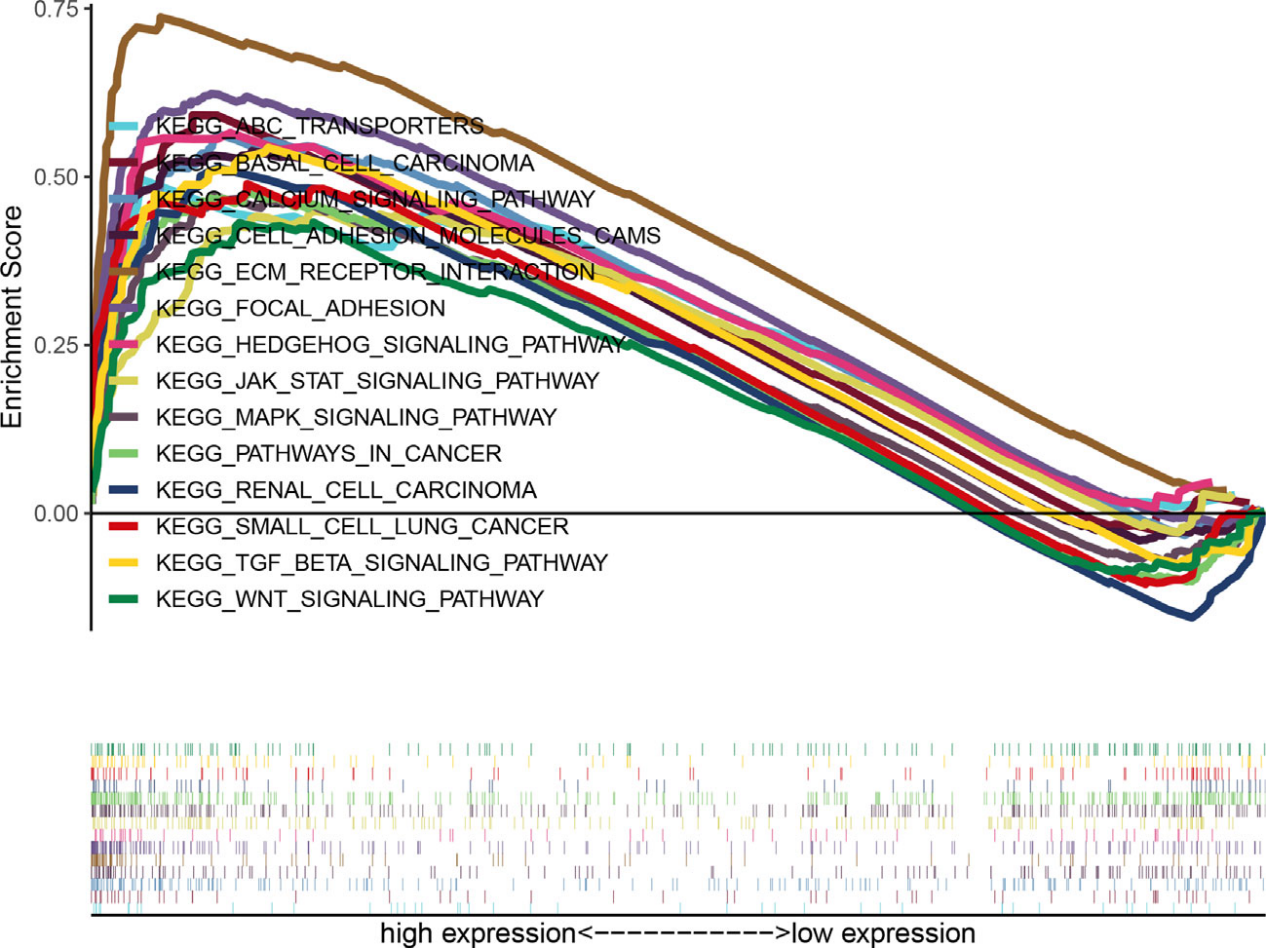
Enrichment plots of multiple signaling pathways from GSEA. Significantly enriched signaling pathways were ECM receptor interaction, Calcium signaling pathway, Focal adhesion, Basal cell carcinoma, Hedgehog signaling pathway, MAPK signaling pathway, TGF-β signaling pathway, Pathway in cancer, Cell adhesion molecule, Renal cell carcinoma, JAK-STAT signaling pathway, ABC transporters, Small cell lung cancer, Wnt signaling pathways. GSEA, gene set enrichment analysis.

According to the median value of GUCY1A2 expression, data were divided into high and low expression sets, and we screened related signaling pathways by GSEA. Based on the NES, FDR Q-value and nominal *P*-value, significantly enriched signaling pathways were selected. There were fourteen enriched and cancer-related signaling pathways: ECM receptor interaction, calcium signaling pathway, focal adhesion, basal cell carcinoma, Hedgehog signaling pathway, MAPK signaling pathway, TGF-beta signaling pathway, pathway in cancer, cell adhesion molecule, renal cell carcinoma, JAK-STAT signaling pathways, ABC transporter, small cell lung cancer, and Wnt signaling pathways (**Table 3** and **Figure 6**).

**Table 3:** Enrichment plots from gene set enrichment analysis.

Gene set name	NES	NOM *p*-value	FDR *q*-value
KEGG_ECM_RECEPTOR_INTERACTION	2.184	0.000	0.004
KEGG_CALCIUM_SIGNALING_PATHWAY	2.139	0.000	0.006
KEGG_FOCAL_ADHESION	2.128	0.000	0.005
KEGG_BASAL_CELL_CARCINOMA	1.927	0.004	0.023
KEGG_HEDGEHOG_SIGNALING_PATHWAY	1.871	0.004	0.034
KEGG_MAPK_SIGNALING_PATHWAY	1.851	0.002	0.032
KEGG_TGF_BETA_SIGNALING_PATHWAY	1.851	0.008	0.030
KEGG_PATHWAYS_IN_CANCER	1.834	0.004	0.032
KEGG_CELL_ADHESION_MOLECULES_CAMS	1.770	0.023	0.049
KEGG_RENAL_CELL_CARCINOMA	1.745	0.012	0.051
KEGG_JAK_STAT_SIGNALING_PATHWAY	1.692	0.006	0.065
KEGG_ABC_TRANSPORTERS	1.686	0.012	0.065
KEGG_SMALL_CELL_LUNG_CANCER	1.650	0.031	0.077
KEGG_WNT_SIGNALING_PATHWAY	1.578	0.028	0.106

## Discussion

Soluble guanylate cyclase has been used in the treatment of cardiovascular diseases such as pulmonary hypertension. Increasing attention has been given to its role in cancer. NO/sGC/cGMP signaling is an important pathway for regulating vascular function, cognition and many other physiological activities. Due to the complex role of NO/SGC/cGMP signaling pathway, it has become a hot issue in cancer research. As an intermediate link, sGC plays an indispensable role in this process. Studies have found differences in the expression of different subunits of sGC in cancer, but there is no research on the expression level of the GUCY1A2 gene encoding the α2 subunit in GC and its prognostic significance.

In this study, we analyzed the differential expression of GUCY1A2 in GC and its significance as a prognostic factor. Moreover, we screened the related enriched signal pathways to understand the mechanism by which GUCY1A2 regulates the development of GC. First, we analyzed the expression of GUCY1A2 in GC tissues and adjacent noncancerous tissues using RNA seq data in TCGA database and found that GUCY1A2 was highly expressed in GC. Next, we performed qRT-PCR and a meta-analysis to verify the high expression of GUCY1A2 in GC, and our findings were consistent with the results of the bioinformatics assay. Moreover, the expression of GUCY1A2 was upregulated with increasing histological grade and T stage. The above findings suggested that GUCY1A2 plays a role in promoting the development of GC. Logistic regression analysis showed that high expression of GUCY1A2 was significantly associated with histological grade (OR = 1.858 for poor vs. well or moderate, *P* = 0.004) and T stage (OR = 3.389 for T3 vs. T1, *P* = 0.025; OR = 3.422 for T4 vs. T1, *P* = 0.028). Kaplan-Meier curves indicate that the prognosis of GC patients with high GUCY1A2 expression is poorer than that of patients with low expression. The same result was obtained by analyzing in the Kaplan-Meier Plotter database. Further subgroup analysis was performed to evaluate the prognostic value of GUCY1A2. It was found that GC patients with high expression of GUCY1A2 in the subgroup of older than 60 years, well or moderate, and T3/4 stage had a poor prognosis. Univariate analysis suggested that GUCY1A2 and some clinicopathological parameters, such as age, pathological stage, and TNM stage, may predict poor prognosis. Multivariate analysis further validated that GUCY1A2 was an independent prognostic factor. Finally, we utilized GSEA to identify the signaling pathways related to GUCY1A2 in GC. The results suggested that ECM receptor interaction, calcium signaling pathway, focal adhesion, basal cell carcinoma, Hedgehog signaling pathway, MAPK signaling pathway, TGF-beta signaling pathway, pathway in cancer, cell adhesion molecule, renal cell carcinoma, JAK- STAT signaling pathways, ABC transporter, small cell lung cancer, and Wnt signaling pathways were correlated with the progression of GC. The ECM is an important part of the tumor microenvironment (TME), which promotes tumor growth and metastasis by affecting physiological functions such as signal transduction, epithelial mesenchymal transition (EMT), and angiogenesis (22, 23). The calcium signaling pathway is related to the proliferation, migration, invasion and formation of drug-resistant cancer cells (24–26). Focal adhesions are closely related to the ECM, which jointly regulates the migration and invasion of cancer cells (27, 28). TGF-β is the main inducer of EMT (29), immune escape and stimulation of metastasis during cancer progression (30). In addition, the TGF-β signaling pathway plays contradictory roles in different stages and cancers. For example, in early stage of breast cancer TGF-β is inhibiting tumor progression, while in advanced stage it plays a role promoting cancer (31, 32). The Hedgehog signaling pathway is activated in cancer and affects tumor development by maintaining and promoting the phenotype of cancer stem cells, stimulating EMT and metastasis (33, 34). Cell adhesion molecules mediate the contact and interaction between cells or between cells and the extracellular matrix and participate in various physiological activities, such as cell recognition, signal transduction, growth and differentiation (35, 36). Moreover, these molecules promote cancer migration and invasion through angiogenesis and destroy the integrity of epithelial cells (37). JAK-STAT participates in the process of internal immune regulation (38). Its imbalance affects tumor growth and development by promoting angiogenesis, regulating the tumor-related matrix and affecting immune escape (39, 40). The ABC transporter removes a variety of chemotherapeutic drugs from the cell, leading to multidrug resistance (MDR) of cancer cells and reducing the effect of chemotherapy. The Wnt signaling pathway and MAPK signaling pathway have been shown to be upregulated in a variety of cancers, and they are involved in regulating the occurrence and development of cancer (41, 42). The above findings provide ideas to explore the carcinogenic and cancer-promoting molecular mechanisms of GUCY1A2, suggesting that GUCY1A2 is involved in the development of GC by regulating various cancer-related molecular signaling pathways.

Our study also has some limitations. First, the number of tumor tissues in the TCGA database was significantly higher than the number of normal tissues used as a control. Second, only the differences in GUCY1A2 mRNA expression level were analyzed, and protein level and direct molecular mechanism were not explored in depth. Finally, the sample size of qPCR assay was relatively small.

In summary, we analyzed the gene expression data of the online database TCGA and found that the expression of GUCY1A2 was higher in GC than in adjacent noncancerous tissues. The same results were obtained in qRT-PCR experiments and GEO database validation. Univariate and multivariate Cox analyses assessed the prognostic significance of GUCY1A2 in GC. Finally, we used GSEA to identify related enriched signaling pathways and preliminarily analyzed the molecular mechanism of GUCY1A2 involvement in gastric carcinogenesis and development. This is the first study to investigate the prognostic value of GUCY1A2 in GC. This study provides a partial basis for screening prognostic biomarkers in GC, but the prognostic value of GUCY1A2 in GC still needs to be explored and validated by more clinical trials and population studies.

## Data Availability Statement

Publicly available datasets were analyzed in this study. This data can be found here: https://portal.gdc.cancer.gov/.

## Ethics Statement

The studies involving human participants were reviewed and approved by the Ethics Committee of Zhongda Hospital, Southeast University. The patients/participants provided their written informed consent to participate in this study.

## Author Contributions

XC conceived and designed the general idea of the study, and analyzed the data. XL performed the experiments, prepared the figures and tables, and wrote the manuscript. XH and RX collected clinical samples, and conducted statistical analysis. LW and YS downloaded and screened the online data. XS directed and supervised the study. All authors contributed to the article and approved the submitted version.

## Funding

This work was supported by the National Natural Science Foundation of China (81472940).

## Conflict of Interest

The authors declare that the research was conducted in the absence of any commercial or financial relationships that could be construed as a potential conflict of interest.

## Supplementary Material

Supplementary Figure 1 Enrichment plots from GSEA. **(A)** ECM receptor interaction; **(B)** Calcium signaling pathway; **(C)** Focal adhesion; **(D)** Basal cell carcinoma; **(E)** Hedgehog signaling pathway; **(F)** MAPK signaling pathway; **(G)** TGF-β signaling pathway; **(H)** Pathway in cancer; **(I)** Cell adhesion molecule; **(J)** Renal cell carcinoma; **(J)** JAK- STAT signaling pathway; **(L)** ABC transporters; **(M)** Small cell lung cancer; **(N)** Wnt signaling pathways. GSEA, gene set enrichment analysis.
