# Supplementary figures and images for: Overexpression of GUCY1A2 Correlates With Poor Prognosis in Gastric Cancer Patients
Source: Front Oncol. 2021 May 25;11:632172. doi: 10.3389/fonc.2021.632172 (PMC8185334; doi:10.3389/fonc.2021.632172)

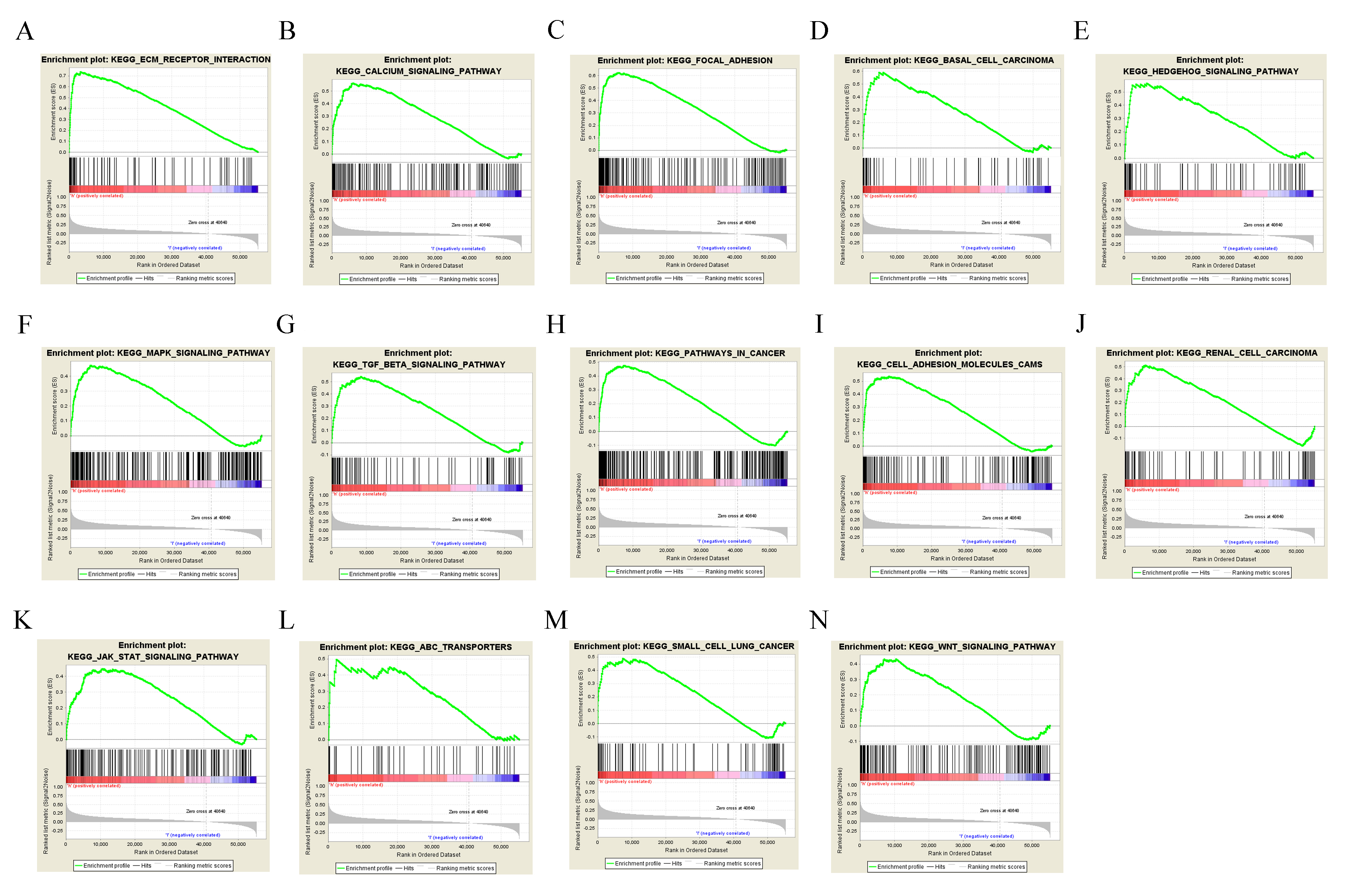

Supplement: Supplementary Figure 1 — Enrichment plots from GSEA. (A) ECM receptor interaction; (B) Calcium signaling pathway; (C) Focal adhesion; (D) Basal cell carcinoma; (E) Hedgehog signaling pathway; (F) MAPK signaling pathway; (G) TGF-β signaling pathway; (H) Pathway in cancer; (I) Cell adhesion molecule; (J) Renal cell carcinoma; (J) JAK- STAT signaling pathway; (L) ABC transporters; (M) Small cell lung cancer; (N) Wnt signaling pathways. GSEA, gene set enrichment analysis. [file Image_1.jpeg]
