# Supplementary material for: Overexpression of GUCY1A2 Correlates With Poor Prognosis in Gastric Cancer Patients
Source: Front Oncol. 2021 May 25;11:632172. doi: 10.3389/fonc.2021.632172 (PMC8185334; doi:10.3389/fonc.2021.632172)
Supplement: Supplementary file 2 [file Table_1.docx]

**Table S1 Clinical characteristics of GC patients from TCGA database**

| Characteristics | Variable | Patients (443) | Percentages (%) |
| --- | --- | --- | --- |
| Age | <60 years | 132 | 29.80 |
|  | ≥60 years | 306 | 69.07 |
|  | Unknow | 5 | 1.13 |
| Gender | Male | 285 | 64.33 |
|  | Female | 158 | 35.67 |
| Histological grade | G1 | 12 | 2.71 |
|  | G2 | 159 | 35.89 |
|  | G3 | 263 | 59.37 |
|  | GX | 9 | 2.03 |
| Pathological stage | Ⅰ | 59 | 13.32 |
|  | Ⅱ | 130 | 29.35 |
|  | Ⅲ | 183 | 41.31 |
|  | Ⅳ | 44 | 9.93 |
|  | Unknow | 27 | 6.09 |
| T | T1 | 23 | 5.19 |
|  | T2 | 93 | 20.99 |
|  | T3 | 198 | 44.70 |
|  | T4 | 119 | 26.86 |
|  | TX | 10 | 2.26 |
| N | N0 | 132 | 29.80 |
|  | N1 | 119 | 26.86 |
|  | N2 | 85 | 19.19 |
|  | N3 | 88 | 19.86 |
|  | NX | 17 | 3.84 |
|  | Unknow | 2 | 0.45 |
| M | M0 | 391 | 88.26 |
|  | M1 | 30 | 6.77 |
|  | MX | 22 | 4.97 |
| Vital status | Alive | 290 | 65.46 |
|  | Death | 153 | 34.54 |

*Data are presented as No. (%). GC, gastric cancer. T, tumor stage; N, node; M, metastasis.*
