# Supplementary material for: Overexpression of GUCY1A2 Correlates With Poor Prognosis in Gastric Cancer Patients
Source: Front Oncol. 2021 May 25;11:632172. doi: 10.3389/fonc.2021.632172 (PMC8185334; doi:10.3389/fonc.2021.632172)
Supplement: Supplementary file 3 [file Table_2.docx]

**Table S2 Information of elected GEO datasets**

| GEO datasets | Year | Country | Platform | Sample size (GC) | Sample size (Non-GC) |
| --- | --- | --- | --- | --- | --- |
| GSE13195 | 2009 | China | GPL5175 | 25 | 25 |
| GSE13911 | 2008 | Italy | GPL570 | 38 | 31 |
| GSE26899 | 2016 | USA | GPL6947 | 96 | 12 |
| GSE27342 | 2011 | USA | GPL5175 | 80 | 80 |
| GSE29272 | 2013 | USA | GPL96 | 134 | 134 |
| GSE33335 | 2012 | China | GPL5175 | 25 | 25 |
| GSE37023 | 2012 | Singapore | GPL96 | 112 | 39 |
| GSE54129 | 2017 | China | GPL570 | 111 | 21 |
| GSE63089 | 2014 | China | GPL5175 | 45 | 45 |
| GSE64591 | 2015 | USA | GPL570 | 63 | 31 |
| GSE65801 | 2015 | China | GPL14550 | 32 | 32 |

*GEO, Gene Expression Omnibus; GC, gastric cancer.*
